# Dipole-Induced Inversion of Spin-Dependent Charge Transport through α‑Helical Peptide-Based Single-Molecule Junctions

**DOI:** 10.1021/jacs.5c10892

**Published:** 2025-09-30

**Authors:** Albert C. Aragonès, Monica Varese, Kavita Garg, Wenzhu Kuang, Qiankun Wang, Ernest Giralt, Vladimiro Mujica, Rafael Gutierrez, Gianaurelio Cuniberti, Luis Puerta, Ismael Díez-Pérez

**Affiliations:** Δ Department of Chemistry, Faculty of Natural, Mathematical and Engineering Sciences, King’s College London, SE1 1DB London, United Kingdom; ◊ Institute for Research in Biomedicine (IRBBarcelona), The Barcelona Institute of Science and Technology (BIST), 08028 Barcelona, Spain; Θ Department of Inorganic and Organic Chemistry, 16724 University of Barcelona, 08028 Barcelona, Spain; Π Department of Molecular Chemistry and Materials Science, Weizmann Institute of Science, 76100 Rehovot, Israel; †† Division of Agriculture, University of Arkansas, Don Tyson Center for Agricultural Sciences, 1371 W. Altheimer Drive, Fayetteville, Arkansas 72704, United States; † Institute for Materials Science and Max Bergmann Center of Biomaterials, Dresden University of Technology, 01062 Dresden, Germany; ‡ 7864 Arizona State University, School of Molecular Sciences, PO Box 871604, Tempe, Arizona 85287-1604, United States; ∇ Departament de Ciència de Materials i Química Física, Institut de Química Teòrica i Computacional, University of Barcelona (UB), Marti i Franquès 1, 08028 Barcelona, Spain; ¶ Dresden Center for Computational Materials Science (DCMS), TU Dresden, 01062 Dresden, Germany

## Abstract

We report on a remarkable phenomenon of interfacial electric dipole inversion coupled to changes in atomic spin densities that translates into the enantiomeric inversion of electron spin-dependent conductance in a 2-terminal single-molecule junction, consisting of a 22 amino acid chiral α-helical peptide sequence connecting two metal electrodes. This phenomenon is conventionally associated with the Chirality-Induced Spin Selectivity (CISS) effect and how it induces spin polarization and spin filtering of the electron transport along the main peptide axis. Here, the inversion of the spin-dependent charge transport behavior is achieved by keeping constant the chiral symmetry of the junction while inverting the direction of the internal electrical dipole moment running along the main helical peptide axis. Using a spinterface model, in which the electrode-molecule injection barriers are dependent both on the electric dipole and magnetic spin moment, we have been able to rationalize the current pattern as arising from a surface dipole inversion and changes in the atomic spin densities in atoms located within the peptide backbone. Both experimental and computational results show that the observed electric dipole-induced spin-dependent transport inversion in the chiral peptide junction links to an inversion in the spin-dependent resistance due to the combined effect of the helical-based CISS effect and the electrode/molecule spinterface.

## Graphical abstract

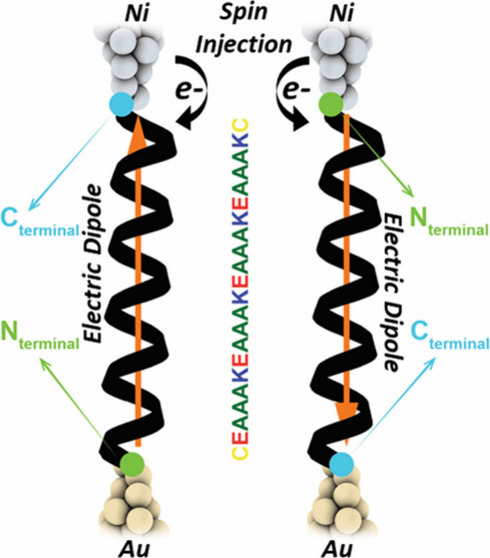

## Introduction

The ability to spin-polarize a transient flow of electrons traveling through a chiral organic medium has been demonstrated in a wide range of platforms, ranging from high energy photoemission^1–3^ to low energy tunnelling electron transport and photoinduced electron transfer measurements.^4–10^ The phenomenon has been vastly observed across broad spatial scales, with numerous examples observed at the nano and single-molecule levels^4,11,12^ and also associated with the magnetic behavior of chiral molecule/electrode interfaces.^13^ Molecular junctions brings opportunities to study the effect in a more controlled setup to address possible microscopic mechanisms at a high level of resolution. First discovered by Naaman and collaborators,^14^ the phenomenon of spin-polarization of a transient current through a chiral medium has been coined as the Chirality-Induced Spin Selectivity (CISS) effect and, since its inception, a number of different theoretical frameworks have tried to provide a description of the underlying physical mechanisms behind it.^15^ It is now well-established that, in addition to the breaking of space-inversion symmetry associated with chirality, the inclusion of spin orbit interaction and the breaking of time-reversal symmetry^16^ are essential ingredients to explain the physics of spin polarization induced by the CISS effect in nonmagnetic interfaces and in the absence of external magnetic fields. In such cases, time-reversal symmetry is broken through the interplay of molecular chirality, spin–orbit coupling, and charge transport. In contrast, in systems involving magnetic electrodes, time-reversal symmetry is explicitly broken by the ferromagnetic order of the substrate. In molecular junctions, the description of spin polarization effects also requires the explicit inclusion of spinterface effects associated with changes in the spin density at the Fermi energy of the surface atoms of the electrode, induced by changes in the interfacial electric dipole moment. These “spinterface” effects have been recently highlighted as an essential ingredient in the regulation of the measured spin-polarization, particularly in electrodes with topological spin textures.^4,17^ The latter has been observed in high resolution photoemission^18^ and single-molecule transport experiments,^4,12^ and recent theoretical proposals support the key role of the spinterface in enabling CISS.^19–23^ At the same time, we need to stress the fact that purely spinterface models cannot explain essential experimental results of the molecular length and temperature dependence of charge transport in molecular junctions.^24–27,48^ Also, an important recent article^8^ demonstrates that spin-polarization in photoinduced electron transfer can occur even without the presence of electrodes. These results clearly indicate that a comprehensive description of the CISS effect in a molecular junction requires at least the inclusion of both spinterface *and* molecular effects. Additional factors involving electron–electron correlations,^28^ the interaction of electrons with vibrational degrees of freedom,^29–33^ and nonadiabatic coupling effects^34^ may also play a role in controlling the spin polarization. Recent measurements of surface potential of a chiral peptide monolayer on a ferromagnetic surface under opposite magnetization directions demonstrate that the spin density at the electrode/peptide interface is different for two different peptide enantiomers.^35,36^ All the above evidence the active role of the electrode/molecule spinterface in the CISS-related spin polarization of the transient electric current crossing the molecular junction. Here we show how the spin density at the molecule/electrode interface correlates with the charge density associated with the interfacial electric dipole moment. This is yet another evidence that in analyzing the response of chiral molecules to electromagnetic fields, the magnetic and electric polarizabilities are intrinsically coupled, as has become clear from a recent analysis of the connection between the chiro-optical response and the spin polarization.^37^

In this work, we synthesize two exact peptide sequences bearing inverted electrical dipole orientations along its main helical axis. The peptides were prepared by solid-state peptide synthesis of a helical sequence (*normal* sequence)^38,39^ and its *retro*-synthesized (*retro* sequence) homologous (Figure 1(a) and (b), respectively). We use a spin-polarized version of the STM break-junction approach^4,23^ to characterize single-molecule charge transport of the *normal* and *retro* peptide sequences under opposite electron spin injections. In this approach, an individual peptide strand is trapped between a Au surface and a magnetically polarized STM Ni probe (Figure 1(a) and (b)). Surprisingly, the observed spin-dependent conductance of the *normal* and *retro* sequences for the *same* enantiomer displays the same spin-selective behavior as the one observed when comparing across the two enantiomers of the same *normal* sequence, i.e., when comparing charge transport results both horizontally and vertically in main Figure 3. In other words, the peptide dipole moment inversion and the peptide enantiomeric substitution have both the same impact on the observed spin-dependent charge transport behavior. We have performed DFT calculations to explore the nature of the interfacial dipole in junctions consisting of two gold electrodes, and a combination of a nickel tip and a gold electrode, mimicking our STM junction arrangement. The computational results suggest that the inversion in the molecular electric dipole translates into an inversion in the spin density at specific atoms of the peptide chain, and hence, into the injection probability inversion of the two spin components, which indicates that the spin-dependent charge transport associated with the CISS effect depends strongly on the orientation of molecular dipole moments in the chiral molecular bridge. Our findings highlight the crucial role of the molecular dipole inversion in modulating spin conductance and regulating CISS, both through the chiral molecular backbone and through the peptide/electrode spinterface. This offers a unified picture that integrates the CISS effect with the interfacial dipole-modulated spin polarization.

**Figure 1:**
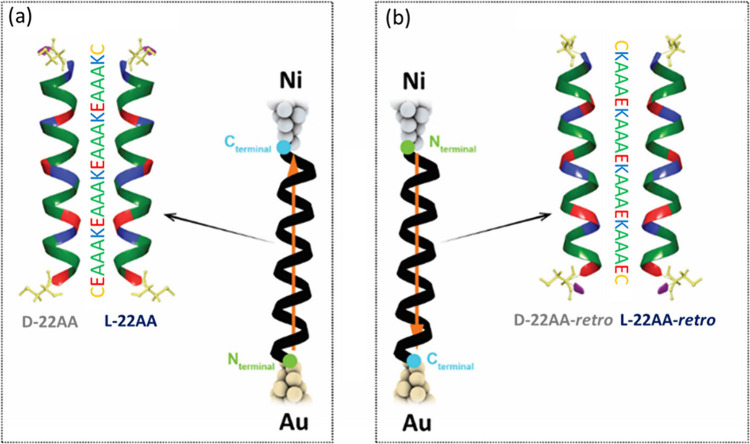
Schematic representation of the alpha helical peptide sequences investigated in this work. D- and L-enantiomers of the *normal* and *retro* peptide sequences are shown in (a) and (b), respectively. The sequence of the peptides is the same (side-by-side) both for the *normal* and *retro* sequences. The representations of the molecular junctions measured including the *normal* (22AA) and the *retro* (22AA-*retro*) sequences, respectively, are also shown, and the orange arrows inside the junction indicate the direction of the molecular electric dipoles with respect to the junction.

## Results and Discussion

### Synthesis of the *normal* and *retro* α-Helical Peptide Sequences

The synthesis of the 22AA peptide analogues (Table 1) was carried out using standard solid phase peptide synthesis (SPPS) by taking into account the high tendency toward epimerization of cysteine (see more details in the Supporting Information (SI) section 1). Cysteine is prone to epimerization both via oxazolone formation during the activation of the C-terminus carboxylic acid (as most other amino acids), and by direct deprotonation during its attachment to the resin and upon prolonged or repeated exposure to a base, i.e. during peptide elongation via fluorenylmethyloxycarbonyl (Fmoc). To avoid the side reaction that typically occurs during the synthesis of C-terminal cysteine peptides via Fmoc SPPS, a glycine residue (G) was added in the peptide sequence as the first amino acid. The synthesis of all peptides used in this study (Table 1) were conducted by SPPS using microwaves assisted technology. The N-terminus was acetylated manually at the end of the peptide assembly by treatment of the peptide resin with a mixture of acetic anhydride and N,N-Diisopropylethylamine. Finally, the peptides were cleaved from the resin, with concomitant removal of the side chain protecting groups. After that, the peptides were purified by reversed-phase high-performance liquid chromatography (RP-HPLC) giving the pure product (SI Figure S1). Note that L- or D-22AA-*retro* in Table 1 denotes the same L- or D-22AA peptide where the sequence has been synthesized in an inverted order.

**Table 1:** Sequence of All 22AA Peptide Analogues Synthesized^a^

	*Chirality*	Peptide name	Peptide sequence
***22AA-normal***			
**1.**	***L***	L-22AA	Ac-**C(SH)**EAAAK​EAAAK​EAAAK​EAAAK**C(Acm)G**-CONH_2_
**2.**	***D***	D-22AA	Ac-**C(SH)**EAAAK​EAAAK​EAAAK​EAAAK**C(Acm)G**-CONH_2_
***22AA-retro***			
**3.**	***L***	L-22AA-*retro*	Ac-**C(Acm)**KAAAE​KAAAE​KAAAE​KAAAE**C(SH)G**-CONH_2_
**4.**	***D***	D-22AA-*retro*	Ac-**C(Acm)**KAAAE​KAAAE​KAAAE​KAAAE**C(SH)G**-CONH_2_

a Acm = Acetamidomethyl. Amino acids in the sequences are expressed in standard notation.

The conformational behavior in solution of the *normal* L- and D-22AA peptides as well as their *retro* analogues were investigated by circular dichroism (CD), which provides information about the global secondary structure of the peptides. The CD spectra of both 22AA and 22AA-*retro* acquired in phosphate buffer (NaP) and 2,2,2-trifluoroethanol (TFE) evidenced the α-helical structure (SI Figure S2), with two characteristic minima at 208 and 222 nm, characteristic of the α-helical secondary structure.^40^ Both D-22AA and D-22AA-*retro* show equivalent CD spectra (SI Figure S2 (a) and (b)), and display an inverted profile compared to that of their L counterparts as expected. The helical content slightly increased in the presence of TFE for both L- and D-peptide analogues (SI Figure S2). The inversion of the dipole direction in the *retro* versions did not have any effect on the optical activity, which shows that when the *retro* and homologous versions are aligned by sequence, they essentially present the same chemistry but with inverted C-, N-termini, i.e., with opposite built-in electrical dipole moment running along the main helix axis.

### Characterization of the Peptide-Functionalized Electrode Surfaces

The target peptide is first adsorbed on a Au electrode surface profiting from the free thiol group at either the C-or the N-terminus (see Table 1 and Methods section for details) prior to the formation of molecular junctions in the STM. We have characterized the peptide-functionalized Au surface by polarization-modulation infrared reflection absorption spectroscopy (PM-IRRAS) to ascertain the preservation of the α-helical secondary structure of the peptide layer upon adsorption on the metal surface and its tilt angle with respect to the surface for both the *normal* and *retro* analogues. Previous PM-IRRAS characterization has shown no spectral differences between two α-helical peptides layers on metal surfaces each composed by the corresponding D- and L-enantiomers.^41^ Therefore, we have here compared both *normal* and *retro* peptide versions of the l-enantiomer only. Amide I (1662 cm^–1^) and II (1545 cm^–1^) IR features are clearly identified for both the *normal* and *retro* peptides (see Figure 2a), which are characteristics of the α-helical secondary structure, indicating the preservation of the α-helical conformation in both peptide layers. The molecular tilt angle was subtracted from the ratio of the intensities of both amide I and II peaks,^42^ leading to a tilt angle value of ca. 42° versus the surface for both peptide versions (Figure 2b), in agreement to previous examples of α-helical peptides layers chemisorbed on a metal surface.^43^ Previous works have shown a good agreement of the extracted tilt angles from PM-IRRAS when compared to those deduced from thickness measurements by scratching experiments using atomic force microscopy^43^ and from the magnetization per molecule using SQUID magnetometry.^42^ Negligible changes were observed among the two *normal* and *retro* homologous versions of the peptides (Figure 2a), concluding that both molecular layers have essentially the same packing and tilt angle when adsorbed onto the metal surface. This consistency enables us to investigate the spin conductance of the peptides under comparable conditions. Since the free thiol group of the terminal cysteine (Cys) residues are located at the N- and C-termini for the *normal* and *retro* sequences, respectively, the electrical dipole moment orientation in the two peptide layers will be oppositely pointing away and toward the electrode surface, respectively.

**Figure 2:**
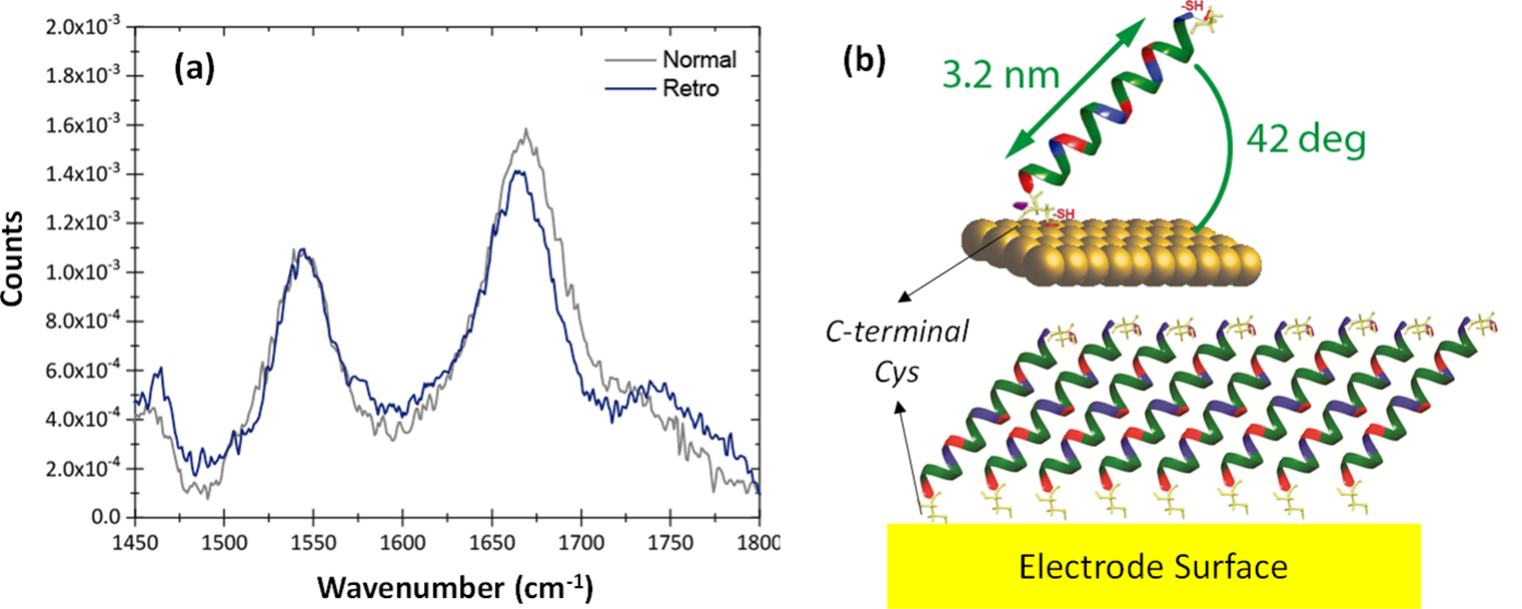
(a) PM-IRRAS spectra for the *normal* and *retro* L-22AA peptide layers on the Au electrode surface. (b) Schematic representation of the peptide-based layer displaying a 42° tilt angle with respect to the surface as suggested by intensity ratio of both Amide I and II peaks in (a).

### Characterization of Single-Peptide Charge Transport

We use a spin-polarized version of the STM break-junction approach^4,8^ to form and electrically characterize the spin-dependent charge transport of single peptide junctions. Briefly, a magnetically prepolarized Ni STM probe is brought in and out to close proximity with the peptide layer deposited on the Au electrode. A constant sample bias voltage difference of +0.1 V is applied between the Au surface and the Ni tip. The current is recorded while the STM tip is retracted away from the surface. When a peptide gets stably trapped (chemically bound via the thiol termini) between the two electrodes, a plateau is observed in the individual current trace displaying the quantized conductance of the molecular junction (insets of Figures 3a-d). We use home-designed algorithms, including those based on clustering methods,^44^ to select the traces displaying plateau features (typically ∼1000 out of >5000 of total individual pulling traces per experiment) and then accumulate the selected ones in the same conductance histogram for its statistical analysis. Gaussian fits of the prominent peaks in the histograms provide the most probable conductance values (peak maxima) and their conductance dispersion (peak width) for the studied molecular junction. The measurements are performed under opposite Ni magnetization directions along the main molecular junction axis.^4^ Magnetizations in different directions other than the main junction axis result in inappreciable spin-dependent effects in the charge transport through the peptide (SI Figure S6). Figure 1 summarizes the four molecular junctions analyzed in this work; the L-,D-enantiomers of the *normal* sequence (Figure 1a), whose electric dipole points away from the Au bottom electrode, and the L-,D-enantiomers of the *retro* sequence (Figure 1b), whose electric dipole points toward the Au bottom electrode. Note that thanks to the *retro* synthesis, the sequence read from top to bottom in both peptides junctions is identical (Figure 1).

**Figure 3:**
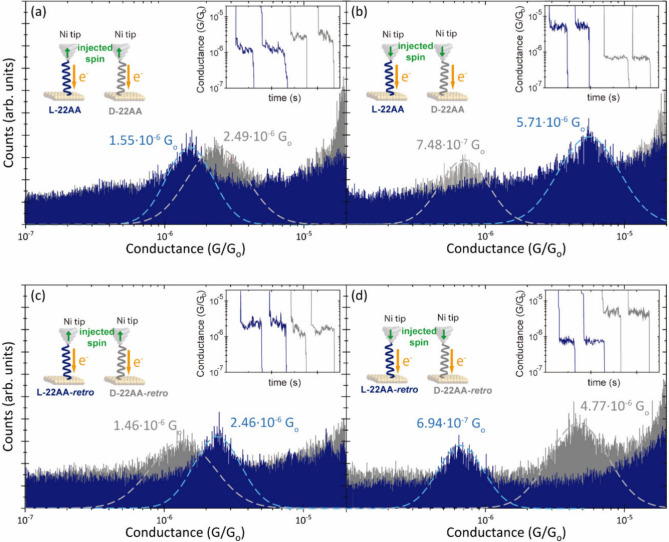
Semilog conductance histograms of the STM break-junction data accumulating >1000 pulling traces for (a-b) L- and D-22AA peptides under injected spin up (left) and down (right). Panels (c-d) show equivalent results for the L- and D-22AA-*retro* peptides. The applied sample bias was set to +0.1 V defining electron injection from tip to sample, indicated by the orange arrow. Insets display representative individual current versus pulling traces used to generate the conductance histograms.

Figure 3 summarizes the results of all single-peptide transport measurements proposed in Figure 1, i.e., the two L-,D-enantiomers of both *normal* and *retro* sequences, each measured under both up and down directions of the Ni STM tip magnetization. The density of states of Ni near the Fermi level reveals that electron injection from a Ni tip predominantly involves minority-spin carrier electrons.^4,45,46^ Thus, in our case the electron spin carriers (green arrows in Figure 3) upon Ni tip magnetization represent injection of the minority spin carriers. Figures 3a and 3b correspond to the peptide conductance results for the 22AA peptides measured for both directions of the Ni magnetization. Similar to our previous results on a homologous peptide sequence,^4^ we observe four different sets of conductance values for all four different scenarios. In the present peptide design, no terminal alkane tails are introduced here for anchoring purposes,^4^ and the peptide is directly flanked by two terminal Cys residues as part of the same sequence. The results show remarkable spin-dependent charge transport in these systems, with a maximum factor of roughly 7 difference in conductance (Figure 3b) for a given Ni magnetization direction between the l- and d-enantiomers. Similar conductance values would be expected for opposite spin magnetizations injected into the opposite enantiomers (compare L- and D-conductance and *vice versa* values from Figures 3a and 3b, respectively); injecting spin down in the l-enantiomer should yield an equivalent outcome as injecting spin up in the d-enantiomer in terms of spin-scattering capabilities. Instead, a factor of ∼2 difference in conductance is observed between the two magnetization directions for the opposite L­(D)- and D­(L)-isomers in both Figures 3a and 3b. Such effects have been previously ascribed to an interfacial magnetization at the peptide/Au interface (spinterface),^19,20,49,50^ which imposes an additional magnetoresistance stage in the molecular device enabling differentiation between opposite L- and D-spin scatterings.^4^ Related to the above, Waldeck et al. have recently measured a ∼40 mV shift in the work function of a Ni/chiral peptide interface under opposite magnetization directions along the surface normal,^35,36^ which suggests possible differences in the charge injection energy barriers at the ferromagnet/peptide interface for the two different spin orientations. The latter could potentially lead to different spin-dependent conductance under the same magnetization conditions in the peptide junction, but a quantitative study connecting these two phenomena is yet to be conducted. Figures 3c and 3d show the results for the analogous measurements on the 22AA-*retro* peptides using the same color legend and same sequence of measurements (see schemes in Figures 3c and 3d insets). The results show that the inversion of the internal electric field orientation of the peptide generates a flip in the order of the spin-dependent conductance for each l- and d-enantiomer; for a given injected spin orientation, the more spin-dependent conductive enantiomer in the *normal* sequence (Figures 3a and 3b) is now the less one in the *retro* one (Figures 3c and 3d). The inversion of electron spin selectivity by a chiral structure upon molecular dipole moment inversion has been suggested previously and ascribed exclusively to the chiral backbone.^51–53^ Our results indicate that the inversion of the internal electric field along the peptide helical structure results in flipping the selected spin carrier in the chiral helical structure, resulting in changes in the spin-dependent resistance due to the combined action of both the helical structure (CISS) and the peptide/electrode interface (spinterface) stage.

### First-Principle Calculations of the Spin and Charge Densities under Opposite Electrical Dipole Directions in the Peptide

Previous work on peptides demonstrated that an inversion of the internal dipole moment of the probed chiral structure results in an inversion of the spin-dependent charge transport behavior across the chiral molecular layer.^52–55^ We have now performed Density-Functional-Theory (DFT) calculations to rationalize how the inversion of the peptide molecular dipoles impacts the spin anisotropy direction at a peptide/Au interface. It has been shown that the nature of the atomic linker in a molecule/electrode interface influences the observed spinterface via changes in the electrostatic potential profile, the magnitude of the injection barrier and the spin density of states (DOS) at the electrode surface.^45,56^ The latter is particularly important because it affects the resulting final spin-polarization of the electric currents crossing the molecular junctions. As described in our previous work,^4^ the low bias voltage conductance within the linear regime can be qualitatively understood within the context of the Landauer model, modified to include an interfacial potential that is capable of altering the spin-dependent DOS at the Fermi level of either a magnetized nickel tip or a gold electrode. Here, we try to establishing a correlation, based on DFT calculations, between the interfacial electric dipole introduced by the peptide helical structure and the spin densities in a peptide/Au junction (Figure 4), that provides a physical explanation for the observed inversion of the spin polarization of the same enantiomer in its *normal* and *retro* sequences (top to bottom flip of conductance in Figure 3).

**Figure 4:**
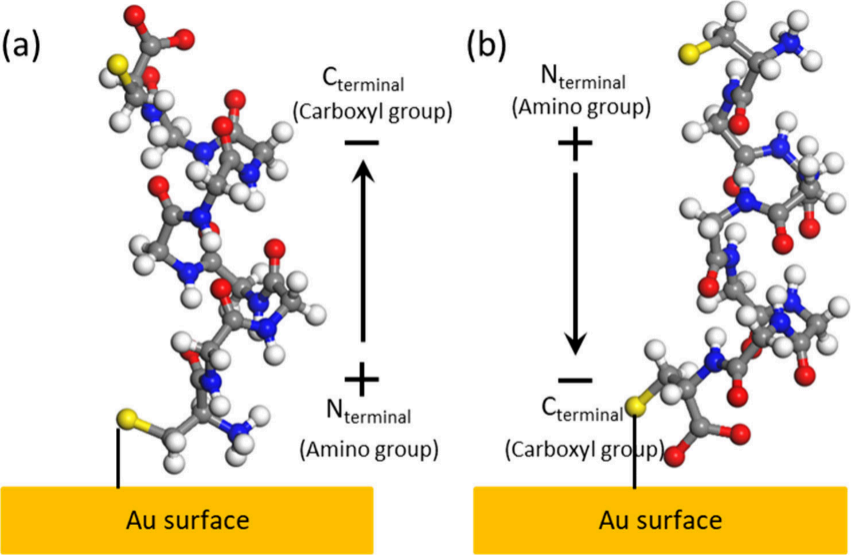
Simplified model for a 10 amino acid helical sequence with axial electric dipole moments pointing in opposite directions toward/from a Au(111) surface: (a) *Normal* peptide orientation with the positive dipole end pointing toward the metallic substrate (N-terminus at the Au surface). (b) *Retro* peptide with the negative dipole end pointing toward the metallic substrate (C-terminus at the Au surface). Color codes for the chemical elements are hydrogen (white), carbon (gray), nitrogen (blue), oxygen (red), and sulfur (yellow).

Details of the DFT calculations are provided in SI section 2. The relevant results included here pertain to the behavior of the Au/peptide interface, where the correlation between the spin and charge densities, determining the magnetic and electric dipole moments, are basically responsible for the observed experimental behavior. The Ni/molecule interface does not exhibit important changes for the *normal* and *retro* peptides.

To perform the computational modeling, a simplified helical peptide model composed of eight l-Gly units and two Cys residues at each end was used. The peptide was oriented perpendicularly to the Au surface (Figure 4). In the first case (Figure 4a), the peptide’s positive pole is positioned closer to the Au surface, homologous to the experimental 22AA-*normal* system (Figure 1a). In the second case (Figure 4b), the peptide’s negative pole is closer to the surface, simulating the 22AA-*retro* sequence orientation (Figure 1b).

The changes in interfacial charge densities reported in the SI Table S1 accounts for the inversion of the interfacial dipole moment indicated in the SI Table S2. In fact, the calculated (z-component) of the dipole moments for the *normal* (*retro*) L sequence; +13.01 D (−5.83 D), describe a significant electric dipole inversion while preserving chiral symmetry. It is also important to stress that the spin density changes occur essentially in the peptide atoms, which are not the nearest neighbors to the gold surface, indicating that there is a significant difference between the interfacial charge and spin redistribution arising from the molecular dipole inversion. This type of spin polarization is similar in its nature to what occurs with coated gold nanoparticles,^57^ where depending on the nature of the adsorbate, an onset of a broken symmetry singlet (BSS) state arises. These BSS is ultimately responsible for the appearance of magnetic behavior in gold nanoparticles, as well as the chiral discrimination associated with magnetic exchange interactions.^58^

Based on these results, we can attempt to formulate a transport model picture for the molecular junction studied here. As suggested by Aragonès et al.,^4^ a qualitative explanation of the observed behavior of the spin-dependent conductance of L- and D-peptides can be achieved by combining the spin (or helicity) selectivity of the helical peptide – resulting from the CISS effect – with an emergent spinterface at the Au–S contact region, i.e., the presence of a quasi-chemical potential describing spin and charge imbalance at this interface. We now generalize the model by Aragonès et al. to include the dipole inversion effect. Since we are only interested in the linear response regime without including many-body effects, we will work within the Landauer theory framework, where the key quantity is the (spin-dependent) conductance obtained from the quantum mechanical transmission function of the molecular junction. We first define two descriptors to label the transmission function to be introduced later on:

1 $$\chi =\frac{\mathrm{s⃗}\cdot \mathrm{p⃗}}{\left|\right|\mathrm{s⃗}\left|\right|\;\left|\right|\mathrm{p⃗}\left|\right|},\delta =\frac{\mathrm{D⃗}\cdot \mathrm{n̂}}{\left|\right|\mathrm{D⃗}\left|\right|}$$

The quantity χ = ±1 defines the electron helicity, i.e., the projection of the electron’s spin on the propagation direction given by its momentum *p⃗*. The helicity can be used, for a given propagation direction, to describe the spin (or helicity) filtering capability of the helical molecule. The second index δ = ±1characterizes the orientation of the molecular dipole vector *D⃗* with respect to the helical axis (keeping the lab frame as reference), which is described by the unit vector *n̂*, which is perpendicular to the Au surface (we do not consider here the peptide tilting): δ = ±1 for parallel (*normal*) orientation and δ = −1 for antiparallel (*retro*) orientation. For the transport properties, we need to introduce also the standard spectral densities of the Au and Ni electrodes, given by

2a $$\Gamma_{Au}\left(E\right)=\underset{k}{\sum }\left|V_{Au,k}{|}^{2}\delta \left(E-E_{k}\right)\approx \right|V_{Au}{|}^{2}\rho_{Au}\left(E\right)$$

2b $$\Gamma_{Ni}^{\sigma }\left(E\right)=\underset{k}{\sum }{\left|V_{Ni,k}^{\sigma }\right|}^{2}\delta \left(E-E_{k}^{\sigma }\right)\approx {\left|V_{Ni}\right|}^{2}\rho_{Ni}^{\sigma }\left(E\right)$$

These quantities describe both the electronic structure of the electrodes as well as the details of the electrode-molecule interaction (via the *V*_*Au(Ni),k*_ -matrix elements). In the previous equations, we have assumed that the coupling matrix elements *V*_*Au(Ni),k*_ are energy- and spin-independent, so that the densities of states *ρ*_*Au*_ (*E*) = ∑_*k*_ δ­(*E* – *E*_*k*_) and Ρ_*Ni*_^σ^ (*E*) = ∑_*k*_ δ­(*E* – *E*_*k*_^σ^) of the corresponding Au and Ni electrodes, respectively, were introduced. Moreover, only their values at the Fermi energy will be considered later, since we are looking at the Landauer conductance. The index σ = ±1 (or ↑,↓) labels the type of spin in the ferromagnetic Ni electrodes, which will be injected into the helical molecule by changing the Ni magnetization direction. It acts thus as an external control variable, but it is not the key quantity determining the observed experimental behavior of the conductance in Figure 3. Exploiting the Dyson equation for the Green’s function of the molecule and assuming a weak coupling between the helical molecule and the electrode-thiol subsystem (the thiol groups are considered as part of an extended interface), we can approximately write the spin-dependent transmission function through the system in the following way:^59,60^

3 $$T_{\delta ,\chi }^{\sigma \sigma \prime }\left(E_{F}\right)=\frac{{\left|V_{Au}\right|}^{2}\rho_{Au}\left(E_{F}\right)}{{\left(\Delta_{S}^{\chi }\right)}^{2}+{\left(\Gamma_{Au}/2\right)}^{2}}|G_{\delta ,\chi }^{\sigma \sigma \prime }\left(E_{F}\right){|}^{2}\frac{{\left|V_{Ni}\right|}^{2}\rho_{Ni}^{\sigma \prime }\left(E_{F}\right)}{{\left(\Delta^{\sigma \prime }\right)}^{2}+{\left(\Gamma_{Ni}^{\sigma \prime }/2\right)}^{2}}$$

We have introduced an effective onset energy difference to the Fermi level Δ_S_^χ^ associated with the Au–S contact of the form: Δ_*S*_^χ^ = *E*_*F*_ – [*E*_*S*_^0^ + (1 + sgn­(χ))­(*U*/2)]. If we assume that the Au–S is fully spin polarized with, let us say, spin up, then this expression means that an energy penalty *U* is paid to transfer a spin-up to the Au electrode (χ=+1 when assuming the propagation direction to be positive from the Ni contact to the Au contact), since a singlet configuration is energetically preferred. Similarly, Δ^σ^ is the energy difference between the Fermi level and a spin-dependent position of an effective level on the Ni interface. All electronic and spin-dependent information about the helical peptide is encoded in *G*_δ,χ_^σσ′^ (*E*_*F*_), whose explicit expression we do not further specify. If spin-flip processes can be approximately neglected (σ = *σ* ^*′*^), the spin-dependent current at low temperatures and low applied voltages can be written as

4 $$I_{\delta ,\chi }^{\sigma }=\frac{e}{h}\int_{E_{F}+e\mathrm{sgn}\left(\delta \right)\mu_{D}}^{E_{F}+eV+e\sigma \mu_{s-ch}}dET_{\delta ,\chi }^{\sigma }\left(E\right)\approx \frac{e}{h}T_{\delta ,\chi }^{\sigma }\left(E_{F}\right)\left(eV+\sigma \mu_{s-ch}-\mathrm{sgn}\left(\delta \right)\mu_{D}\right)=\frac{e^{2}}{h}T_{\delta ,\chi }^{\sigma }\left(E_{F}\right)\left(1+\frac{\mu_{eff}^{\delta ,\sigma }}{V}\right)V$$

In eq 4, we have introduced in the integration limits the (quasi) chemical spin-charge potential *μ*_*s*__–*ch*_ associated with the molecule/Ni interface^61^ and the influence of the inversion of the electrical dipole moment via a term at the Au/molecule interface sgn­(δ)*μ*_*D*_ of the peptide when going from the 22AA-*normal* form to the 22AA-*retro* form. Hence, for a given magnetization direction in the Ni electrode, there is an additional tuning of the effective chemical potential Μ_*eff*_^δ,σ^ related to the peptide dipole direction. In summary, the conductance of the Au-peptide-Ni junction *g*_δ,χ_^*M*^, written as

5 $$g_{\delta ,\chi }^{\sigma }=g_{0}T_{\delta ,\chi }^{\sigma }\left(E_{F}\right)\left(1+\frac{\mu_{eff}^{\delta ,\sigma }}{V}\right)$$

encodes phenomenologically the different experimental scenarios presented in Figure 3.

Taking into account all the above-mentioned considerations, Figure 5 schematically represents the spin-dependent transport picture of the studied peptide junctions. The corresponding experimental values of the conductance (Figure 3) have been organized in ascending order from left to right in the central G axis. For the 22AA (top panel), consistent with our previous observations,^4^ the two higher conductance values (r+R and r+r in Figure 5 top panel) correspond to the spin orientation preferred by the peptide helical structure, i.e., the L-22AA favors spin-down transport while D-22AA favors spin-up transport. The highest conductance (r+r) reflects the spin favored by both the peptide and the spinterface stages. In contrast, the two lower conductance values (R+R and R+r in Figure 5 bottom panel) correspond to spin orientations that are less favorable for the chiral peptides, indicating higher resistance for spin-up transport in the l-enantiomers and *vice versa* for the d-enantiomers. The lowest conductance observed for the d-enantiomer (R+R) corresponds to a spin state unfavorable to both the molecule and the spinterface. On the other hand, in the case of 22AA-*retro* (bottom panel), where the internal electric field is reversed, the order of the conductance of the four peptides is completely inverted, i.e., under the same spin orientation injection conditions, the most conductive peptide in the *normal* sequence becomes the least conductive in the *retro* sequence and *vice versa*. Previous photoemission and magnetic-conductive AFM (mc-AFM) measurements also demonstrate that the sign of the spin-polarized current reverses with the orientation of the molecular dipole on the electrode.^51–53^ For instance, Carmeli et al. attributed an inversion of spin selectivity of the ejected photoelectrons crossing a chiral peptide monolayer to the change of the internal magnetic field, which is determined by both the direction of the molecular dipole moment and the handedness of the helical peptide.^51^ Our results further confirm this behavior: the inversion of the internal molecular electric field flips the preferred spin component in the chiral molecular backbone (red arrows in Figure 5), thus corroborating the major role of the chiral peptide in the spin filtering mechanism across the molecular junction. Specifically, the l-enantiomer, which favors spin-down transport in the 22AA configuration (r+r in Figure 5 top panel), now favors spin-up transport in the 22AA-*retro* (R+R in Figure 5 bottom panel) and the opposite for the d-enantiomer. From the current experimental results, we cannot completely rule out that the inversion of spin selectivity upon electrical dipole reversal could be due to a change in the dominant charge carrier type (electron vs hole),^62^ as a shift in the vacuum energy level induced by the interfacial dipole could alter the relative energy level alignment of the molecular frontier orbitals (i.e., LUMO, HOMO) with respect to the electrode Fermi levels, potentially favoring hole conduction in one configuration and electron conduction in the other. The latter could, in principle, lead to the observed inversion in spin selectivity. However, based on previous Kelvin probe measurements on molecular layers of similar peptide systems,^51^ the measured change in surface work function upon dipole reversal is approximately 0.3 eV (for a molecular dipole moment of ∼10 D), which is generally small to significantly alter the alignment to the extent required to switch from electron to hole transport. Notwithstanding, while the latter awaits experimental verification, the observed conductance splitting leading to a four distinct conductance features still requires the introduction of a spinterface stage for a full interpretation of the experimental data.

**Figure 5:**
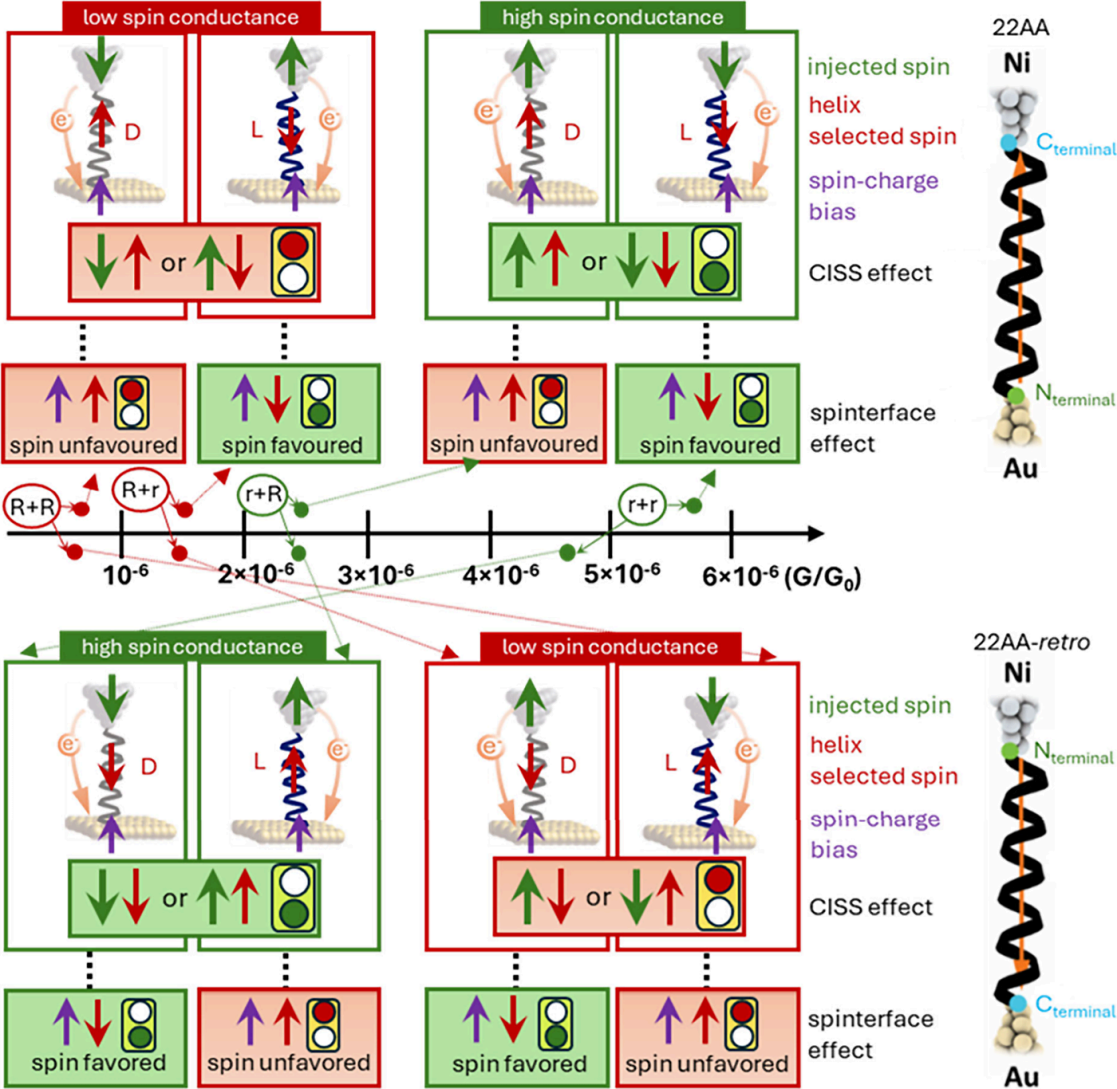
Schematic representation of the spin-dependent charge transport across the Ni-peptide-Au junction denoting the two stages; the spin-filtering process at the chiral helical structure (CISS) and at the spinterface, for both *normal* (22AA) and *retro* version (22AA-*retro*) under opposite Ni magnetization directions. The experimental conductance values (*G*) are indicated in the central axis for all measured peptide junctions. The binary lights signal the magnetoresistance to charge transfer; green is the lower (*r*) and red is the higher (*R*) resistor. The central light refers to the spin selected by the chiral molecule (CISS effect), while the smaller bottom light refers to the spin charge (Spinterface) effect.

Based on our theoretical calculations, we observe that the main inversion of spin density is observed at the atomic constituents of the peptide backbone (see s_i_ values in SI Table S1), contributing to spin flipping and leaving the spinterface effect as a constant spin correction factor in all cases (fixed violet arrow in Figure 5).

## Conclusions

Our work demonstrates that inverting the internal electrical dipole moment of a chiral helical peptide of identical sequence produces an inversion of the spin-dependent charge transport behavior measured in a Au-peptide-Ni molecular junction. We show that the observed flip in the spin-dependent conductance behavior by both L- and D-helices of the peptide, which has been conventionally associated with the CISS effect, occurs in the single-peptide junction due to an inversion of the electron spin preferred at the helical structure (CISS), which leads to changes in the spin-dependent resistance both at the helical peptide and at the spinterface stages. Our results evidence the key role of the molecular dipole moment in the regulation of the CISS effect and reinforces the need of a constant spinterface term in all case studies for a full description of the experimental results. Our calculations show the electric dipole inversion couples to changes in the atomic spin densities in the peptide backbone which, together, mimics the effect, reported in several experiments, of exchanging the two (D, L) enantiomers in the molecular junction. We want to stress out here that we report large spin polarizations (SP) of ∼60%^4^ in long molecular junctions bearing 22+ chiral centers and a chiral secondary structure, which is in striking contrast to recently reported results by Li et al.^47^ on very short (∼1 nm) chiral molecules with barely a single chiral center, reporting absence of spin-dependent conductance. We are currently preparing a work reporting the length-dependent spin SP in the studied peptide sequences, which predicts that for short peptides (>5 residues sequence) the SP values might then be too small to be experimentally measured in a single-molecule junction, and which evidence that the results by Li et al.,^47^ while interesting and valid, are limited to particular cases and that under no circumstances can be generalized to all molecular junctions.

## Methods

### Sample Preparation and STM Measurement Conditions

High-purity Au (111) single crystal disk (10 mm × 1 mm, 99.9999% purity) was obtained from MaTeck (Germany). Prior to measurement, the Au (111) substrate was electropolished to remove residual contamination and annealed using hydrogen flame to achieve a clean surface. The peptides (22AA-*normal* and 22AA- *retro*) were then functionalized on the clean Au (111) electrode surface through self-assembly, covalently bind via a free thiol group at either the N-terminal (for *normal* peptides) or the C-terminal (for *retro* peptides). Cysteine residues at the opposite terminal were protected by an acetamidomethyl (Acm) group to prevent adsorption during the thiol group deposition. To form a high quality of self-assembled monolayer, the Au (111) crystal was incubated in peptide solution (200 μM) in a 6:4 mixture of trifluoroethanol (TFE) and water for 24 h. After the adsorption, the Acm group was removed by immersing the surface in a 15% acetic acid solution with freshly prepared iodine (I_2_) in methanol at 4 °C for 24 h. The surface was then rinsed with Milli-Q water and immersed in sodium thiosulfate (Na_2_S_2_O_3_) solution to stop oxidation. The peptide-modified Au (111) surface was then placed into the cell with 80 μL Milli-Q water/TFE solution to maintain the helical conformation during transport measurements conducted using a scanning tunneling microscope (STM). Data captures were acquired using a NI-DAQmx/BNC-2110 National Instruments (LabVIEW data acquisition System) and analyzed with our LabVIEW code. Our automated selection procedure is based on three main criteria for rejecting traces: (1) excessive noise, characterized by large oscillations; (2) featureless, long, nonexponential decays; and (3) “empty” exponential decays lacking discernible current plateaus. Individual trace histograms are analyzed where plateaus are identified as conductance regions with a high level of counts along a clean exponential background. These criteria ensure the selection of curves, such as those shown in the inset of Figure 3, which exhibit rapid exponential decays with clearly visible plateau features.

Nickel (Ni) tips were prepared by mechanically cutting a polycrystalline Ni wire (99.99%, Goodfellow, UK). The freshly cut Ni tip was electrically insulated with Apiezon wax and magnetically polarized ex- situ by placing it in close proximity to a ±1T NdFeB magnet for a period of 2 h (see Figure S7). As a result, an up- or down-polarized Ni tip was obtained. Due to the weak magnetocrystalline anisotropy of Ni,^63^ the magnetic polarization of the tip is always along the STM tip. To avoid the Ni wire oxidation during the polarization process the prepared Ni electrode was stored in strict anaerobic conditions before use. Once the tip is transfer to the STM cell, it is driven several times toward the Au surface to get a light Au coating that protects the tip apex surface against further oxidation throughout the time frame of all the single molecule transport experiments.

### Polarization Modulation Infrared Reflection–Absorption Spectroscopy (PM-IRRAS) Measurements

For PM-IRRAS measurement target peptide is first adsorbed on a Au electrode. Au-coated (50 nm) P++ doped Si wafers were cleaned by sonicating for 5 min each in acetone and ethanol, followed by UV/ozone treatment for 15 min. Cleaned Au slides/patterned chips were activated by treatment with hot ethanol for 30 min, and dried with N_2_ and immediately transferred to the protein solution and incubated at 4 °C for 1 h. After 1 h, the slides on which the protein was deposited were gently cleaned with H_2_O and dried with N_2_.

PM-IRRAS measurements were performed using a Nicolet 6700 FTIR, at an 80° incidence angle, equipped with PEM-90 photoelastic modulator (Hinds Instruments, Hillsboro, OR) with modulation wavenumber of 1600 cm^–1^ for the amide I and II regions. Raw spectra were smoothed and baseline-corrected by a spline algorithm.

## Supplementary Material



## Notes

Details on the peptide synthesis including solid-phase synthesis method and characterization by UPLC, UPLC-MS and CD. Details on the computational model and methodology. STM conductance experiment controls on perpendicular magnetizations (PDF)
