# Supplementary material for: Dipole-Induced Inversion of Spin-Dependent Charge Transport through α‑Helical Peptide-Based Single-Molecule Junctions
Source: J Am Chem Soc. 2025 Sep 30;147(40):36453–63. doi: 10.1021/jacs.5c10892 (PMC12512102; doi:10.1021/jacs.5c10892)
Supplement: Supplementary file 1 [file ja5c10892_si_001.pdf]

Supplementary Information

Dipole-Induced Inversion of Spin-dependent Charge Transport through α-helical Peptide-based Single-Molecule Junctions

Albert C. Aragonès,[∇] Monica Varese,[◊] Kavita Garg,[Π] Wenzhu Kuang,[Δ] Qiankun Wang,[Δ] Ernest Giralt,[◊ Θ] Vladimiro Mujica,[‡ *] Rafael Gutierrez,[†] Gianaurelio Cuniberti,[†,¶] Luis Puerta,[††] and Ismael Díez-Pérez[Δ*]*

[Δ] Department of Chemistry, Faculty of Natural, Mathematical and Engineering Sciences, King's College London, United Kingdom.

[◊] Institute for Research in Biomedicine (IRBBarcelona), The Barcelona Institute of Science and Technology (BIST), 08028 Barcelona, Spain.

[Θ]Department of Inorganic and Organic Chemistry, University of Barcelona, 08028 Barcelona, Spain.

[Π] Department of Molecular Chemistry and Materials Science, Weizmann Institute of Science, 76100 Rehovot, Israel.

[††] Division of Agriculture, University of Arkansas, Don Tyson Center for Agricultural Sciences, 1371 W. Altheimer Drive, Fayetteville, AR 72704, USA

[†] Institute for Materials Science and Max Bergmann Center of Biomaterials, Dresden University of Technology, 01062 Dresden, Germany.

[‡] Arizona State University, School of Molecular Sciences, PO Box 871604, Tempe, Arizona 85287-1604, USA.

[∇] Departament de Ciència de Materials i Química Física, Institut de Química Teòrica i Computacional, University of Barcelona (UB), Marti i Franquès 1, 08028 Barcelona, Spain.

[¶] Dresden Center for Computational Materials Science (DCMS), TU Dresden, 01062 Dresden, Germany.

Corresponding author: ismael.diez_perez@kcl.ac.uk, vmujica@asu.edu, acortijos@ub.edu

Keywords: *(Bio)Molecular Electronics, CISS, STM break-junction, single-peptide junction, spin filter, electrical dipole moment inversion.*

Table of Contents

Contents:

1. Peptides synthesis and characterization 2
1.1 Solid-phase peptide synthesis (SPPS) 2
1.2 Peptide characterization 3
2. Computational model and methodology 5
3. STM conductance histograms 11
4. References 11

1. Peptides synthesis and characterization

1.1 Solid-phase peptide synthesis (SPPS)

All the peptides were prepared using an automated microwave-assisted peptide synthesiser (Liberty Blue synthesizer, CEM) on a 100 µM scale by using RinkAmide ChemMatrix® resin (with a substitution of 0.54 mmol/g). Prior to attachment of the first amino acid, the resin was swollen in DMF (dimethylformamide) for 1 hour. The coupling protocol used 5 equivalents of each Fmoc-amino acid in DMF (0.2 M) and an activation mixture containing OxymaPure (5 equiv, 1M in DMF) and DIC (5 equiv, 0.5 M in DMF) in DMF. The reaction mixtures were stirred for 3 min at 90 ^{0}C, except for cysteines, which were coupled at 50 ^{0}C for 10 min). The solvents were removed by filtration, and the resin was washed with DMF (3 x 30 s). Fmoc deprotection was carried out using 10% (w/v) piperazine and 0.1 M OxymaPure in a 9:1 mixture of NMP and EtOH (1 min). The N-terminus was acetylated manually at the end of peptide assembly by treatment of the peptide resin with a mixture of Ac_2O (15 eq) and DIPEA (30 eq) in DMF at room temperature for 1 hour.

Peptides were cleaved with concomitant removal of the side-chain protecting groups, by treatment with a mixture of 92.5% (v) TFA, 2,5% (v) TIS, 2.5% (v) H_2O and 2.5% (w/v) DTT at room temperature for 1 hour. After cleavage of the peptides, the solvent was evaporated applying a stream of N_2 and the peptide was precipitated by addition of ice-cold *tert*-butyl methyl

ether. The suspension was centrifuged at 4000 rpm and 4 ^{0}C for 10 min. The ether fraction was discarded and the process repeated up to 3 times. The cleaved peptides were then dissolved in H_2O/MeCN (1:1) with 0.1% TFA and freeze-dried.

Peptides were purified by automated flash chromatography on IscoCombi flash equipment by using a ReadySep C_{18} column. Mobile phase flow rate of 30 mL/min (solvent A: 0.1% TFA in water, solvent B: 0.1% TFA in ACN) and a 40 min linear gradients of ACN in H_2O. Peaks of interest were analyzed by UPLC, pure fractions (>95%, by HPLC analysis) were collected and lyophilized. The final products were analyzed and fully characterized by UPLC and UPLC-MS.

1.2 Peptide characterization

UPLC-MS Analysis. Chromatograms and spectra were recorded on a Waters high class (PDA detector, sample manager FNT and Quaternary solvent manager) coupled to an electrospray ion source ESI-MS Micromass ZQ and using the MassLynx 4.1 software (Waters, Milford, MA). Using a BEH C_{18} column (50 x 2.1 mm x 1.7 μm, Waters). The flow rate was 0.6 mL/min, and MeCN (0.07% formic acid) and H_2O (0.1% formic acid) were used as solvents. Samples were analyzed with positive ionisation: the ion spay voltage was 30 V and the capillary temperature was 1 kV.

UPLC-PDA Analysis. UPLC chromatograms were obtained on an Acquity high class (PDA detector, sample manager FNT and Quaternary solvent manager), using an Acquity BEH C_{18} (50 x 2.1 mm x 1.7 μm) column. The flow rate was 0.61 mL/min and MeCN (0.036% TFA) and H_2O (0.045% TFA) were used as solvents. 2-min linear gradients were used in all cases. Detection was performed at 220 nm.

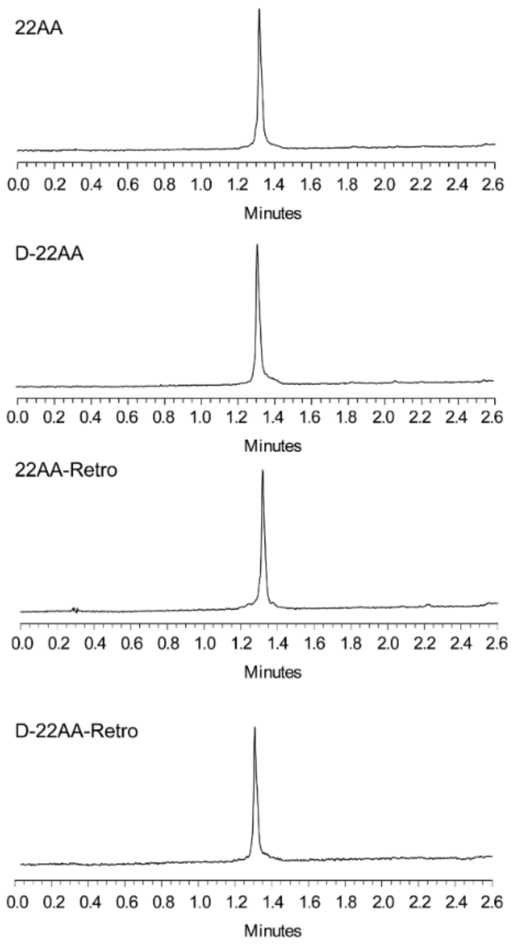

Figure S1. UPLC traces of all peptides described in this paper. UPLC chromatograms are recorded at 220 nm in a 2-min linear gradient from 10 to 40% of MeCN (0.036% TFA) in H_2O (0.045%TFA).

Circular Dichroism (CD) Spectroscopy. Circular dichroism spectra (Figure S2) were recorded using a Jasco 810 UV-Vis spectropolarimeter, equipped with a CDF 426S/426L peltier. Spectra were registered at 25^{0}C in the far UV region from 190 to 280 nm, with a time response of 2 seconds, a scanning speed of 20 nm/min and a step resolution of 0.2 nm. Each spectrum was obtained averaging three scans (within 600 HT voltage range), subtracting contributions from corresponding blanks and converting the signal to mean residue ellipticity in units of deg × cm^2 × $dmol^{-1}$ × res^{-1}. The concentration of peptide was 40 μM, and a 0.1 cm path-length quartz cuvette was used. Spectra were obtained in 10 mM sodium phosphate buffer (pH 7.5) aqueous solution in the absence and in the presence of 20% (v/v) trifluoroethanol (TFE). Spectra were analyzed using the spectral analysis software and smoothed using 'adaptive smoothing' function.

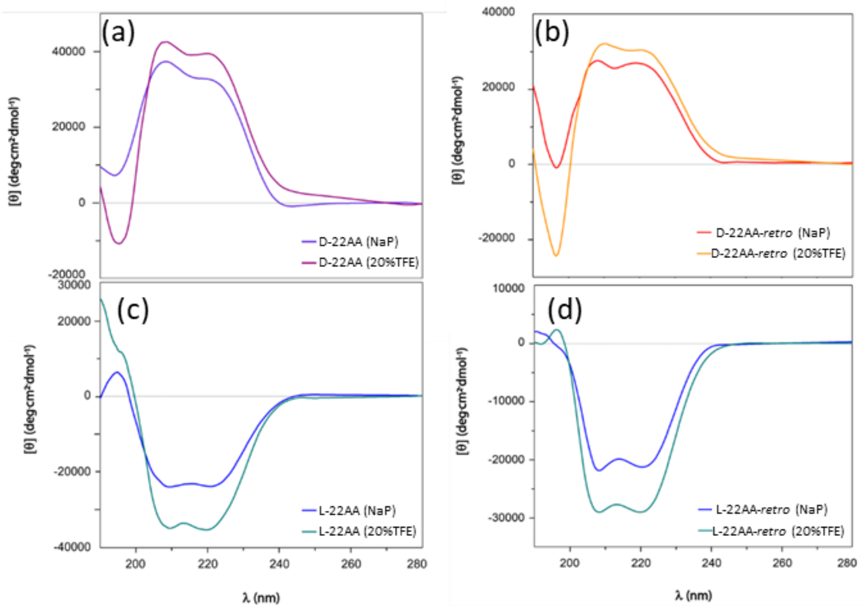

Figure S2. CD spectra of L- and D- 22AA and 22AA-*retro* peptides measured in phosphate buffer (NaP) and 20% TFE. The spectral feature of the D-isomers in (a) and (b) show inverted profile compared to that of L-isomers in (c) and (d).

2. Computational model and methodology

To simulate the structures of the peptides used experimentally, a smaller peptide was employed (Figure S3), which can be oriented in either of its two possible orientations with respect to the surface of the gold electrode. This peptide was constructed with eight units of L-Gly (–HNCOCH2–)$_8$ and two units of cysteine (Cys) at each end. When absorption occurred through the sulfur group near the N-terminal, it represented the absorption of the *normal* peptide, while absorption through the sulfur group near the C-terminal represented the *retro* peptide. To replicate the high dipole moments of the experimental peptides due to their larger size, the molecule in its zwitterionic form was used for all adsorption simulations (Figure S4). The value of the dipole moment[1] clearly indicates that the zwitterionic form of the molecule has a higher dipole moment than its non-ionized form, as the zwitterionic structure has a greater amount of localized charge distributed over a larger distance.

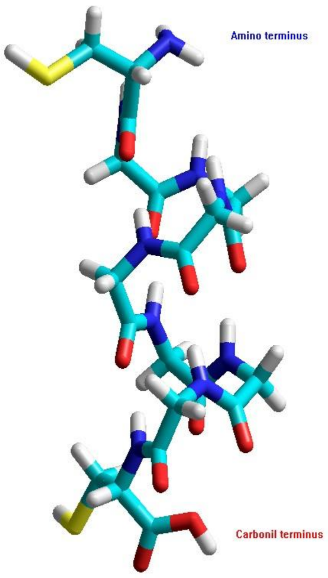

Figure S3. Representation of an alpha-helix structure of 8 glycine (Gly) residues, and two units of cysteine (Cys) at each end, showing the amino (N) and carboxyl (C) terminus.

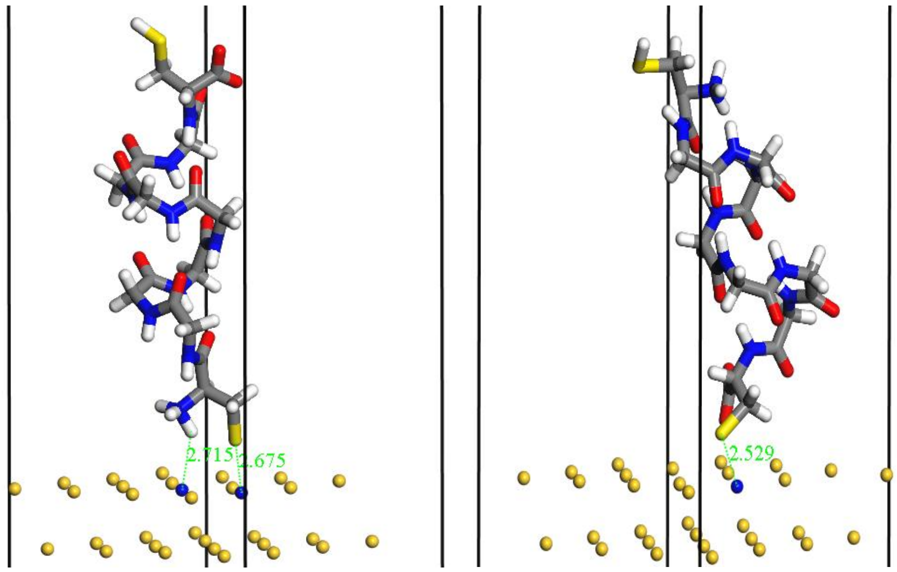

Figure S4. Left panel: *Retro* peptide orientation with the negative dipole end pointing toward the metallic substrate surface (C-terminus at the substrate surface). Right panel: *Normal* peptide with the positive dipole end pointing toward the metallic substrate surface (N-terminus at the substrate surface). Color codes for the chemical elements are as follows: hydrogen (white), carbon (gray), nitrogen (blue), oxygen (red), and sulfur (yellow). Gold atoms are depicted as yellow spheres. The green dashed lines indicate the distances between the atoms at the ends of each line, with the measurements shown in green. The top and bottom of the supercell are not shown to enhance the resolution of the image.

The Au electrode surface that resulted from cutting a (111) face-centered-cubic crystal was modeled as p(4x4) 3D supercells with four layers with a width of 9.81 Å (including 64 Au atoms). The super cell dimensions were: a = b = 11.53 Å, c= 70 Å. Angles (degree) were α=β= 90˚ and γ =120˚. The symmetric slab method used by Kronik *et al.* to avoid the need for dipole corrections was applied in all cases.[2] Further details on the preparation of α-helixes for 3D supercells construction can be found in Refs.[3, 4]

Dispersion corrected Density Functional Theory (DFT-D) calculations were carried out using periodic boundary conditions using the DMol3 (Accelrys)[5] code. The Perdew-Burke-Ernzerhof functional (PBE)[6] functional was combined with the method proposed by Tkatchenko and Scheffler (TS) for DFT-D[7]. In both systems (Figure S4), partial geometry optimization of the peptide-surface electrode was executed using unrestricted DFT with a numeric basis set DNP (UDFT/DNP), where inner electrons were treated using a DFT semi-core pseudopotential. Initially, a total optimization of the entire supercell was performed. However, during the optimization process, the atoms at the ends of the peptide, specifically the amino or carbonyl groups of glycine farthest from the surface, tended to reach an extremely exaggerated distance, greater than 4 Å. Therefore, for those functional groups, the optimization was carried out by keeping them at a fixed distance, close to the expected experimental distance. Some properties of the polypeptide and surface electrode were calculated for the magnetic states, including the Hirshfeld charge and the spin magnitude of the various atoms in the system.

To obtain the magnetic states[3,4] for the peptide-Au surface electrode systems, a spin was added to some atoms of the system before starting the unconstrained SCF calculation. This was done to verify if the SCF magnetic state is maintained at the end, either by maintaining or redistributing the spin. In the referenced paper, a high multiplicity state is initially set, and then it is relaxed to a singlet state through an SCF calculation, thereby obtaining an open-shell singlet.

The classification of the atoms, as shown in Figure S5, was performed as follows: the atoms of the first peptide group following the cysteine residue were labeled O1, C1, and N1; those of the second peptide group as O2, C2, and N2; and those of the third peptide group as O3, C3, and N3.

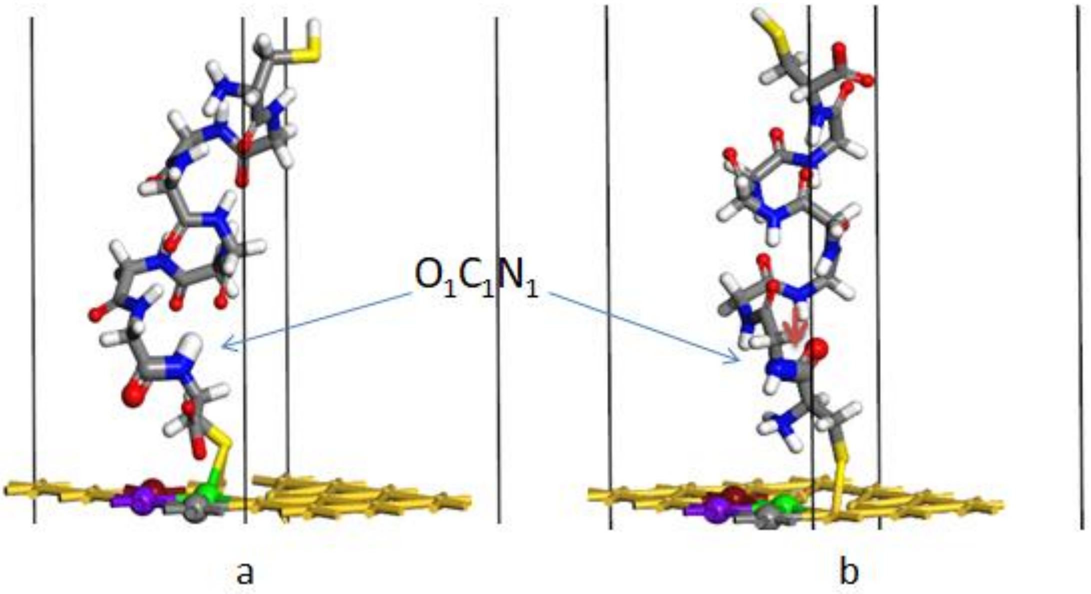

Figure S5. The details of the computational methodology include the representation of the peptide in both its retro and normal sequences. Figure (a) shows the retro sequence, while Figure (b) corresponds to the normal sequence. The illustration highlights the positions of the O1, C1, and N1 atoms in each case. To highlight these atoms, a larger radius has been used in the stick representation. Color codes for the chemical elements are as follows: hydrogen (white), carbon (gray), nitrogen (blue), oxygen (red), and sulfur (yellow). The gold atoms used to calculate their charge and dipole moment are represented as spheres with the following colors: Au61 in purple, Au26 in brown, Au45 in gray, and Au62 in green. The remaining atoms are represented using yellow sticks.

The charge and spin differences, Δq and Δs, respectively, were determined for the same atom i between the *retro*-sequence and the *normal* sequence. For example, for atom i, the charge difference was calculated as $\Delta q = q_i$ (*retro*) – q_i (*normal*). The spin density, s_i, was defined as the difference between the α and β electron populations at atom i (i.e., $s_i = \rho^{\alpha}_i - \rho^{\beta}_i$), as obtained from a Hirshfeld population analysis. Accordingly, the spin difference was calculated as $\Delta s = s_i$ (*retro*) – s_i (*normal*).

Computational analysis based on Hirshfeld population methods reveals notable charge redistribution at the metal–peptide interface, particularly involving Au atoms and the adjacent sulfur atom (Table S1). Despite this, no spin polarization is observed in these atoms for either peptide orientation. Instead, spin-polarized charge transfer is localized on peptide atoms such as

nitrogen, oxygen, and carbon, highlighting the organic fragment as the main contributor to the magnetic response in the system.

Table S1 contains the difference in charge and spin densities for the *normal* and *retro* sequences. The difference in charges is responsible for the inversion of the electric dipole moment, whereas the difference in spin density influences the initial spin population arising from the spinterface effects.

The dipole moment $\vec{\mu}$ was calculated using the expression[1]: $\vec{\mu} = \sum_j q_j \vec{r}_j$, where the q_j are Hirshfeld charges on each atom and $\vec{r}_j$ are the corresponding atomic positions. Since the largest contribution to the dipole moment in all cases arises from its z-component, we only report in what follows the changes of this component, μ_z. For the dipole moment values reported in Table S2, only the gold atoms indicated in Table S1 are considered.

Table S1. The table presents the charge distribution q_i, spin density s_i, as well as the charge difference Δq and spin density difference Δs, for selected atoms in both peptide sequences: *retro* and *normal*. When the spin density is equal to zero, the corresponding entry is left blank. It is important to note that the gold atoms, the sulfur atom closest to the surface, and the atoms belonging to peptide groups with an index lower than 5 all exhibit zero spin density. The sulfur atom included in the table is the one located nearest to the gold surface atoms. Additionally, positive spin density values are shown in blue, while negative values are indicated in red.

**Atom**	**q_i *Retro*-peptide**	**q_i *Normal*-peptide**	**s_i *Retro*-peptide**	**s_i *Normal*-peptide**	**Δq**	**Δs**
Au_{61}	0.0543	-0.0385			0.0928	
Au_{26}	0.0355	-0.0398			0.0753	
Au_{45}	0.0855	-0.0345			0.1200	
Au_{62}	0.1030	-0.0111			0.1141	
S	-0.0602	-0.1929			0.1327	
O_1	-0.2716	-0.2290			-0.0426	
C_1	0.1547	0.1485			0.0062	
H_1	0.1018	0.1353			-0.0335	
N_1	-0.0826	-0.0868			-0.0042	
O_5	-0.2363	-0.2283			-0.0080	
C_5	0.1454	0.1527	0.0001		-0.0073	0.0001

H_5	0.0949	0.0897	0.0017		0.0052	0.0017
N_5	-0.0887	-0.0897	0.0002		0.0010	0.0002
O_6	-0.2444	-0.2624	0.0018	-0.0001	0.0180	0.0017
C_6	0.1441	0.1427	0.0017		0.0014	0.0017
H_6	0.0676	0.0779	0.0056	-0.0001	-0.0103	0.0055
N_6	-0.1141	-0.1017	0.0033		-0.0124	0.0033
O_7	-0.2272	-0.2705	0.0007	-0.0040	0.0433	0.0047
C_7	0.1529	0.1464	0.0048	-0.0006	0.0006	0.0054
H_7	0.0889	0.092	0.0137	-0.0005	-0.0071	0.0142
N_7	-0.0973	-0.0921	0.0052	-0.0071	-0.0005	0.0123
O_8	-0.2878	-0.2166	0.1392	-0.1380	-0.0712	0.2772
C_8	0.0584	0.1496	0.2436	-0.0151	-0.0912	0.2587
H_8	0.0739	0.10150	0.0084	-0.0001	-0.0276	0.0085
N_8	-0.1305	-0.0796	0.0862	-0.0106	-0.0509	0.0968

Table S2. Z-component of the electric dipole moment, perpendicular to the electrode-molecule interface, calculated using the DFT-D method.

Peptide	μ_z(Debye)
Retro	+13.01
Normal	-5.83

3. STM conductance histograms

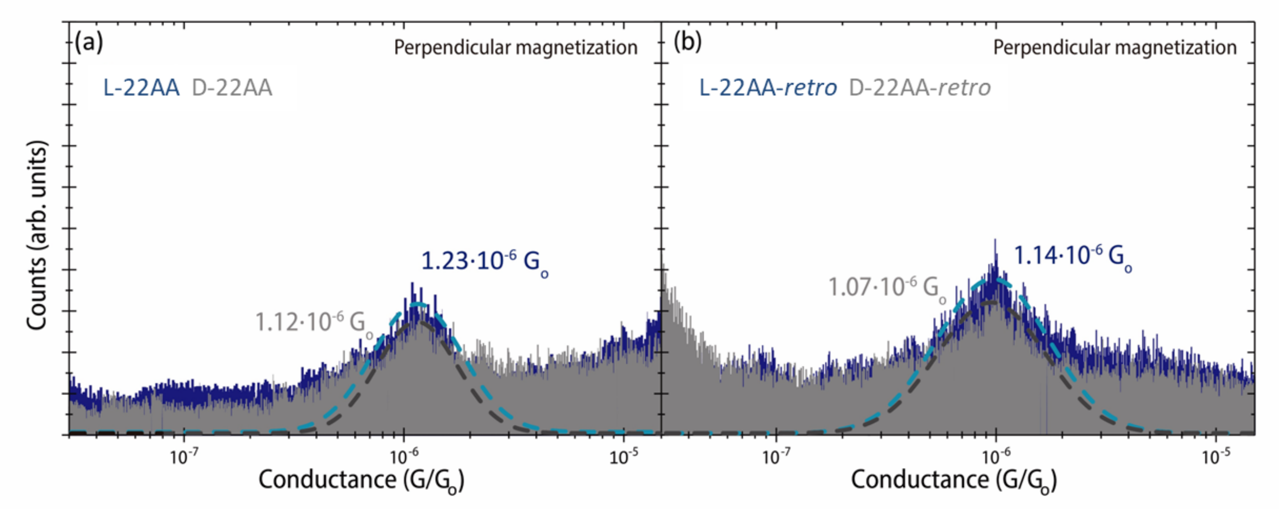

Figure S6. Semilog conductance histograms of the STM break junction data for *normal* (left panel) and *retro* (right panel) peptides, as measured with the Ni tips magnetically polarized perpendicular to the tip axis. the conductance values (peak maxima of the Gaussian fit) of L- and D- isomer peptides for both *normal* and *retro* versions are almost the same.

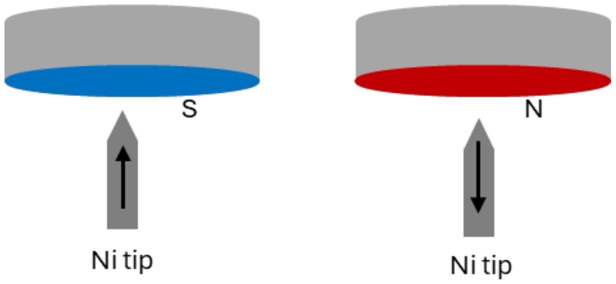

Figure S7. Schematic illustration of the Ni tip magnetized in the up (left panel) and down (right panel) directions by placing the Ni tip electrode in close proximity to the south and north pole of a NdFeB magnet. The arrows within the Ni tip indicate the direction of the magnetic moments.

REFERENCES

(1) Cox, H. *Problems and Solutions to Accompany McQuarrie and Simon's Physical Chemistry*; University Science Books, **1997**.

(2) Natan, A.; Kronik, L.; Shapira Y. Computing surface dipoles and potentials of self-assembled monolayers from first principles. *Appl. Surf. Sci.* **2006**, *252* (21), 7608–7613.

(3) Puerta, L.; Franco, H; Murgich, J.; Gonzalez, C.; Simón-Manso, Y.; Mujica, V. Dipole Orientation and Surface Cluster Size Effects on Chemisorption-Induced Magnetism: A DFT Study of the Interaction of Gold-Thiopolypeptide. *J. Phys. Chem. A* **2008**, *112* (40), 9771–9783.

(4) Puerta, L.; Gonzalez, C.; Franco, H.; Mujica, V. Self-Assembled Monolayers of Thiopolypeptides on Gold Surfaces for the Development of Electronic and Photonic Devices. **2014**, DOI: 10.13140/RG.2.2.35397.60643.

(5) Delley, B. An all-electron numerical method for solving the local density functional for polyatomic molecules. *J. Chem. Phys.* **1990**, *92* (1), 508–517.

(6) Hammer, B.; Hansen, L. B.; Nørskov, J. K. Improved adsorption energetics within density-functional theory using revised Perdew-Burke-Ernzerhof functionals. *Phys. Rev. B* **1999**, *59* (11), 7413–7421.

(7) McNellis, E. R.; Meyer, J.; Reute, K. Azobenzene at coinage metal surfaces: Role of dispersive van der Waals interactions. *Phys. Rev. B* **2009**, *80* (20), No. 205414.
